# Removal of *p16* ^*INK4*^ Expressing Cells in Late Life has Moderate Beneficial Effects on Skeletal Muscle Function in Male Mice

**DOI:** 10.3389/fragi.2021.821904

**Published:** 2022-01-26

**Authors:** Steve D. Guzman, Jennifer Judge, Shahjahan M. Shigdar, Thomas A. Paul, Carol S. Davis, Peter C. Macpherson, James F. Markworth, Holly Van Remmen, Arlan Richardson, Anne McArdle, Susan V. Brooks

**Affiliations:** ^1^ Department of Molecular and Integrative Physiology, University of Michigan, Ann Arbor, MI, United States; ^2^ Department of Musculoskeletal Biology, Institute of Ageing and Chronic Disease, University of Liverpool and MRC-Arthritis Research UK Centre for Integrated Research Into Musculoskeletal Ageing (CIMA), Liverpool, United Kingdom; ^3^ Department of Biomedical Engineering, University of Michigan, Ann Arbor, MI, United States; ^4^ Aging and Metabolism Research Program, Oklahoma Medical Research Foundation, Oklahoma City, OK, United States; ^5^ Department of Physiology, University of Oklahoma Health Sciences Center, Oklahoma City, OK, United States; ^6^ Oklahoma City VA Medical Center, Oklahoma City, OK, United States; ^7^ Department of Biochemistry and Molecular Biology, University of Oklahoma Health Sciences Center, Oklahoma City, OK, United States

**Keywords:** senescence, aging, sarcopenia, muscle atrophy, inflammation

## Abstract

Aging results in the progressive accumulation of senescent cells in tissues that display loss of proliferative capacity and acquire a senescence-associated secretory phenotype (SASP). The tumor suppressor, p16^*INK4A*^, which slows the progression of the cell cycle, is highly expressed in most senescent cells and the removal of p16-expressing cells has been shown to be beneficial to tissue health. Although much work has been done to assess the effects of cellular senescence on a variety of different organs, little is known about the effects on skeletal muscle and whether reducing cellular senescent load would provide a therapeutic benefit against age-related muscle functional decline. We hypothesized that whole-body ablation of p16-expressing cells in the advanced stages of life in mice would provide a therapeutic benefit to skeletal muscle structure and function. Treatment of transgenic p16-3MR mice with ganciclovir (GCV) from 20 to 26 months of age resulted in reduced p16 mRNA levels in muscle. At 26 months of age, the masses of tibialis anterior, extensor digitorum longus, gastrocnemius and quadriceps muscles were significantly larger in GCV-treated compared with vehicle-treated mice, but this effect was limited to male mice. Maximum isometric force for gastrocnemius muscles was also greater in GCV-treated male mice compared to controls. Further examination of muscles of GCV- and vehicle-treated mice showed fewer CD68-positive macrophages present in the tissue following GCV treatment. Plasma cytokine levels were also measured with only one, granulocyte colony stimulating factor (G-CSF), out of 22 chemokines analyzed was reduced in GCV-treated mice. These findings show that genetic ablation of p16^+^ senescent cells provides moderate and sex specific therapeutic benefits to muscle mass and function.

## Introduction

Biological aging is characterized by the progressive accumulation of tissue damage that leads to an overall reduction in both lifespan and healthspan. Unlike congenital diseases that can be attributed to a single gene mutation, the dysfunction and pathologies that accompany aging are a result of abnormalities in many cellular and molecular processes. These include genomic instability, telomere attrition, mitochondrial dysfunction, stem cell exhaustion, loss of proteostasis, and cellular senescence (López-Otín et al., 2013).

The cellular and molecular processes associated with organismal aging contribute to tissue aging in varying degrees and temporal onsets depending on the organ, as well as hereditary and environmental factors (Ferrucci et al., 2010; Ori et al., 2015; Schaum et al., 2020). With skeletal muscle aging, there are several broad clinical and physiological presentations that are associated with frailty. These hallmarks consist of a progressive decline in muscle mass and resultant decrease in strength, termed sarcopenia (Bortz, 2002; Studenski and Pahor, 2009). Currently, the only available and effective interventions for ameliorating age-associated muscle loss are caloric restriction and exercise (Valdez et al., 2010). Thus, there is a critical need to elucidate the mechanisms responsible for age-related muscle atrophy and weakness to develop more effective and broadly applicable treatments for increasing healthspan.

Cellular senescence is characterized by a terminal state of cell growth and has been shown to contribute to the aging process in many different tissues (Di Micco et al., 2021). Upon entering the state of cellular senescence, cells adopt a senescence associate secretory phenotype (SASP), which involves an upregulation of pro-inflammatory cytokine expression and protein secretion that further reinforces the cellular senescence program in cells within the microenvironment (Faget et al., 2019). Reducing cellular senescence appears to improve physical performance in old age (Baker et al., 2016; Xu et al., 2018). Furthermore, repression of cellular senescence through *p16* ^*INK4A*^ silencing and inhibition of reactive oxygen species (ROS) generation in muscle stem cells has been found to improve stem cell quiescence and autophagy (Sousa-Victor et al., 2014; García-Prat et al., 2016). To date there are few studies investigating the impact of cellular senescence directly on age-associated muscle atrophy and weakness.

In the present study, we assessed muscle function in an aged genetic mouse model (p16-3MR) in which *p16* ^*INK4A*^-expressing senescent cells can be eliminated upon treatment with the drug ganciclovir (GCV). Here we show that male p16-3MR mice treated with GCV starting at 20-month of age through to 26-month of age exhibited reduced muscle atrophy and increased force generation in select hindlimb muscles compared with vehicle-treated mice in which p16-expressing cells were not deleted.

## Materials and Methods

### Animals

**FIGURE 1:**
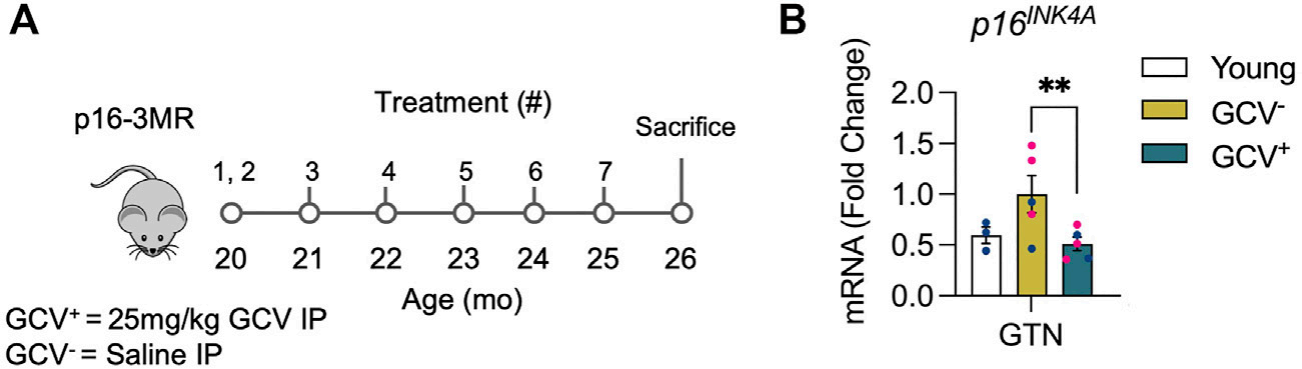
GCV^+^ muscle showed reduced expression of *p16* ^*INK4A*^. **(A)** p16-3MR mice were given ganciclovir (GCV^+^) or vehicle (GCV^−^) *via* intraperitoneal (IP) injection at 20-month of age for 10 days over 2 weeks. Additional 5-day treatments were administered over 1 week in five subsequent months until sacrifice after 6 months. Tissue was harvested at 26-month of age. **(B)** p16 mRNA relative to 18s mRNA was reduced nearly ∼50% in gastrocnemius muscles of GCV^+^ mice compared to saline treated controls (GCV^−^). Bars show the mean ± SEM of three to five mice per group with dots representing data from each individual mouse. Blue and red data points represent male and female mice, respectively. ** Denotes *p* < .001 between groups for each tissue by one-way ANOVA with Tukey post-hoc test.

Aged p16-3MR mice were obtained from the laboratory of Dr. Arlan Richardson at the Oklahoma University Health Sciences Center at ∼19 months of age and adult (4–6 months) C57BL/6 mice were obtained from Charles River Laboratories and served as young adult controls. All mice were housed under specific pathogen-free conditions with ad-libitum access to food and water in the University of Michigan Unit for Laboratory Animal Medicine. p16-3MR mice have been previously characterized and validated (Demaria et al., 2014). These transgenic mice contain a truncated herpes simplex virus 1 (HSV-1) thymidine kinase (HSV-TK) that is driven by the p16^INK4a^ promoter. This design allows for selective ablation of p16 expressing cells upon exposure to the guanosine analog antiviral, ganciclovir (GCV). GCV interacts with HSV-TK and leads to conversion of GCV into a toxic DNA chain terminator resulting in death of non-dividing senescent cells *via* mitochondrial DNA damage and caspase-dependent apoptosis (Laberge et al., 2013; Demaria et al., 2014). To determine the effects of late-life removal of p16-expressing senescent cells on skeletal muscle function, starting at 20 months of age, p16-3MR mice were treated with GCV (25 mg/kg) for five consecutive days during each of the first 2 weeks of the study and then an additional 5 days once per month until the mice reached 25-month of age (Figure 1A). Control p16-3MR mice (GCV^−^) were injected with an equal volume of saline (Figure 1A). At 26 months of age, 4 weeks after last treatment, muscle force generation was measured as described below and hind limb muscles and blood were collected and processed for analysis.

### *In Vivo* and *In Situ* Force Testing

Mice were anesthetized with initial intraperitoneal injections of Avertin (tribromoethanol, 250 mg/kg) with supplemental injections to maintain an adequate level of anesthesia during all procedures. For *in vitro* contractile properties, soleus, and extensor digitorum longus (EDL) muscles were carefully removed from the animal and placed in a horizontal bath containing buffered mammalian Ringer solution (in mM: 137 NaCl, 24 NaHCO_3_, 11 glucose, 5 KCl, 2 CaCl_2_, 1 MgSO_4_, 1 NaH_2_PO_4_, and .025 turbocurarine chloride) maintained at 25°C and bubbled with 95% O_2_-5% CO_2_ to stabilize pH at 7.4. One tendon was tied to a force transducer (model BG-50, Kulite Semiconductor Products, Leonia, NJ) and the other tendon to a servomotor (model 305B, Aurora Scientific, Aurora, ON). Muscles were stimulated by square pulses delivered by two platinum electrodes connected to a high-power biphasic current stimulator (model 701B, Aurora Scientific). Custom-designed software (LabVIEW 2018; National Instruments, Austin, TX) controlled electrical pulse properties and servomotor activity and recorded data from the force transducer. The voltage of pulses was increased, and optimal muscle length (L_o_) was subsequently adjusted to give maximum twitch force (Brooks and Faulkner, 1988). The L_o_ was measured with digital calipers. Muscles were held at L_o_ and subjected to trains of pulses to generate isometric contractions. Pulse trains were 300 ms for EDL muscles and 900 ms for soleus muscles. Stimulus frequency was increased until the maximum isometric force (P_o_) was achieved (Brooks and Faulkner, 1988). Previously established L_f_-to-L_o_ ratios of 0.44 for EDL muscles and 0.71 for soleus muscles (Brooks and Faulkner, 1988) were used to calculate L_f_ for each muscle. The physiological cross-sectional area (CSA) of muscles was determined by dividing the mass of the muscle by the product of L_f_ and 1.06 g/cm^3^, the density of mammalian skeletal muscle. P_o_ was normalized by the CSA to calculate specific P_o_ (sP_o_), as a measure of intrinsic force generating capacity.

Gastrocnemius (GTN) muscle contractile properties were measured *in situ*, as described by Larkin et al. (2011). In anesthetized mice, the whole GTN muscle was isolated from surrounding muscle and connective tissue using great care not to damage the nerve and/or blood vessels during the dissection. A 4-0 silk suture was tied around the distal tendon and the tendon was severed. The animal was then placed on a temperature-controlled platform warmed to maintain body temperature at 37°C. The hindlimb was securely tied to a fixed post with 4-0 monofilament nylon suture at the knee and the foot was clamped to the platform. The distal tendon of the GTN muscle was then tied to the lever arm of a servomotor (model 6650LR, Cambridge Technology). A continual drip of saline warmed to 37°C was administered to the GTN muscle to maintain its temperature. The muscle was activated by stimulation of the tibial nerve using a bipolar platinum wire electrode. Similar to the *in vivo* force testing protocol used for the EDL and TA, the optimal voltage and L_o_ was determined based on the maximal twitch force. The same procedure was then repeated, but rather than activating the muscle *via* the tibial nerve, a cuff electrode was placed around the proximal and distal ends of the muscle for direct muscle stimulation. After force measurements, the GTN was removed trimmed of tendons, blotted and weighed. GTN muscle fiber length (L_f_) was calculated by multiplying L_o_ by 0.45. CSA and sPo were calculated as described above.

EDL, soleus, and GTN muscles were also removed, trimmed, and weighed and all muscles were either snap-frozen for molecular analyses or coated in tissue-freezing medium (Electron Microscopy Sciences) and rapidly frozen in isopentane cooled with liquid nitrogen for histologic analysis.

### Immunofluorescent Imaging and Analysis

Cross-sections (10 μm) were cut from the muscle mid-belly in a cryostat at −20°C and adhered to SuperFrost Plus slides. Prepared slides for immune cell staining were blocked and permeabilized in blocking buffer (5% normal goat serum and 0.2% Triton X-100 in PBS) for 30 min at room temperature followed by overnight incubation at 4°C with primary antibodies. The following day, slides were incubated with Alexa Fluor conjugated secondary antibodies DAPI and mounted using Fluorescence Mounting Medium (Agilent Dako, S302380). A subset of slides was incubated with Alexa Fluor conjugated wheat germ agglutinin (Invitrogen, W32466). Slides prepared for satellite cell staining were lightly fixed with 2% PFA for 5 min and rinsed with 0.1% Triton X-100 in PBS (PBST), then blocked with 10% AffinePure Fab Fragment Goat Anti-Mouse IgG (Jackson ImmunoResearch), 2% bovine serum, and 5% normal goat serum overnight in 4°C. Primary anti-Pax7 (1:10) antibodies were applied to slides for 30 min at room temperature, followed by incubation with Alexa Fluor conjugated secondary antibody and DAPI (1 μg/ml) for 30 min at room temperature. The following primary antibodies (Abs) were used: rat anti-CD68 (Bio-Rad MCA1957), rabbit anti-Laminin (Abcam #7463), and mouse anti-Pax7 (DHSB #Pax7). Fluorescent images were captured using a Nikon A1 confocal microscope. Muscle morphology was analyzed on stitched panoramic images of the entire muscle cross-section by high-throughput fully automated image analysis with the MuscleJ plugin for FIJI/ImageJ (Mayeuf-Louchart et al., 2018). Immune cells and satellite cells were manually counted throughout the entire cross-section and then normalized to tissue area and fiber number as determined by MuscleJ. In all cases, the experimenter was masked to the experimental group.

### Reverse Transcription Quantitative PCR

Frozen muscles were homogenized in Trizol (Thermo Fisher Scientific, Waltham, MA, United States). RNA was extracted and isolated with chloroform separatory extraction and isopropyl alcohol precipitation. The RNA samples were then treated with DNase (Invitrogen). Total RNA was reverse transcribed with SuperScript Reverse Transcriptase III using random hexamers to prime the extension (Thermo Fisher Scientific) to produce cDNA, which was then used for quantitative PCR (RT-qPCR) using SYBR Green qPCR Master Mix (Bio-Rad Laboratories, Hercules, CA, United States), according to the manufacturer’s protocol. Whole muscle gene expression was measured by RT-qPCR on a CFX96 Real-Time PCR Detection System (Bio-Rad, 1855195) in 20 μl reactions of iTaq™ Universal SYBR® Green Supermix (Bio-Rad, #1725124) with 1 μM forward and reverse primers (Table 1) or with TaqMan® probes. Relative mRNA expression was determined using the 2^−ΔΔCT^ method. The following primers/probes were used:

**TABLE 1:** Real-time PCR primers.

***Primers***		
Gene		Sequence (5′-3′)
TNF-α	Forward	ATG​GCC​TCC​CTC​TCA​TCA​GT
	Reverse	TGG​TTT​GCT​ACG​ACG​TGG​G
IL-6	Forward	TCC​GGA​GAG​GAG​ACT​TCA​CA
	Reverse	TTG​CCA​TTG​CAC​AAC​TCT​TTT​CT
AChRα	Forward	GCC​ATT​AAC​CCG​GAA​AGT​GAC
	Reverse	CCC​CGC​TCT​CCA​TGA​AGT​T
MuSK	Forward	ACC​GTC​ATC​ATC​TCC​ATC​GTG​T
	Reverse	CTC​AAT​GTT​ATT​CCT​CGG​ATA​CTC​C
18s	Forward	GCT​TGC​TCG​CGC​TTC​CTT​ACC​T
	Reverse	TCA​CTG​TAC​CGG​CCG​TGC​GTA
*Probes*		
Gene	Assay ID	
p16(INK4a)	Mm00494449_m1 (Thermo Fisher Scientific)	

### Plasma Protein Analysis

Blood samples from p16-3MR mice were collected from the descending aorta while mice were under anesthesia (isoflurane). Blood samples were collected into EDTA coated microcentrifuge tubes and centrifuged at 10,000 g for 10 min at 4°C and plasma samples were collected. Multiplex analysis for cytokines, chemokines and other factors was performed using the Luminex Bio-Plex Pro Mouse Cytokine 23-plex Assay (#M60009RDPD) and measured using the Bio-Plex 200 system (BioRad, Hertfordshire, United Kingdom).

### Statistical Analyses and Data Presentation

Data are presented as the mean ± SEM. Statistical analysis was performed in GraphPad Prism 9. Between-group differences were tested by two-tailed unpaired Student’s t-test (2 groups) or by one-way ANOVA (more than two groups). *p* ≤ .05 was used to determine statistical significance.

## Results

### Reduced p16 Expression in Skeletal Muscle in GCV Treated p16-3MR Mice

To verify successful depletion of p16-expressing cells, quantitative-PCR (qPCR) was performed in skeletal muscle (gastrocnemius muscles). A roughly 50% reduction of p16 expression was found in skeletal muscles of GCV treated mice (GCV^+^) compared to saline treated controls (GCV) (Figure 1). These data show that removal of muscle-resident p16-expressing cells can be achieved with the p16-3MR model.

### Muscle Mass and Force Is Greater in Select Hind Limb Muscles After Removal of p16-Expressing Cells

**FIGURE 2:**
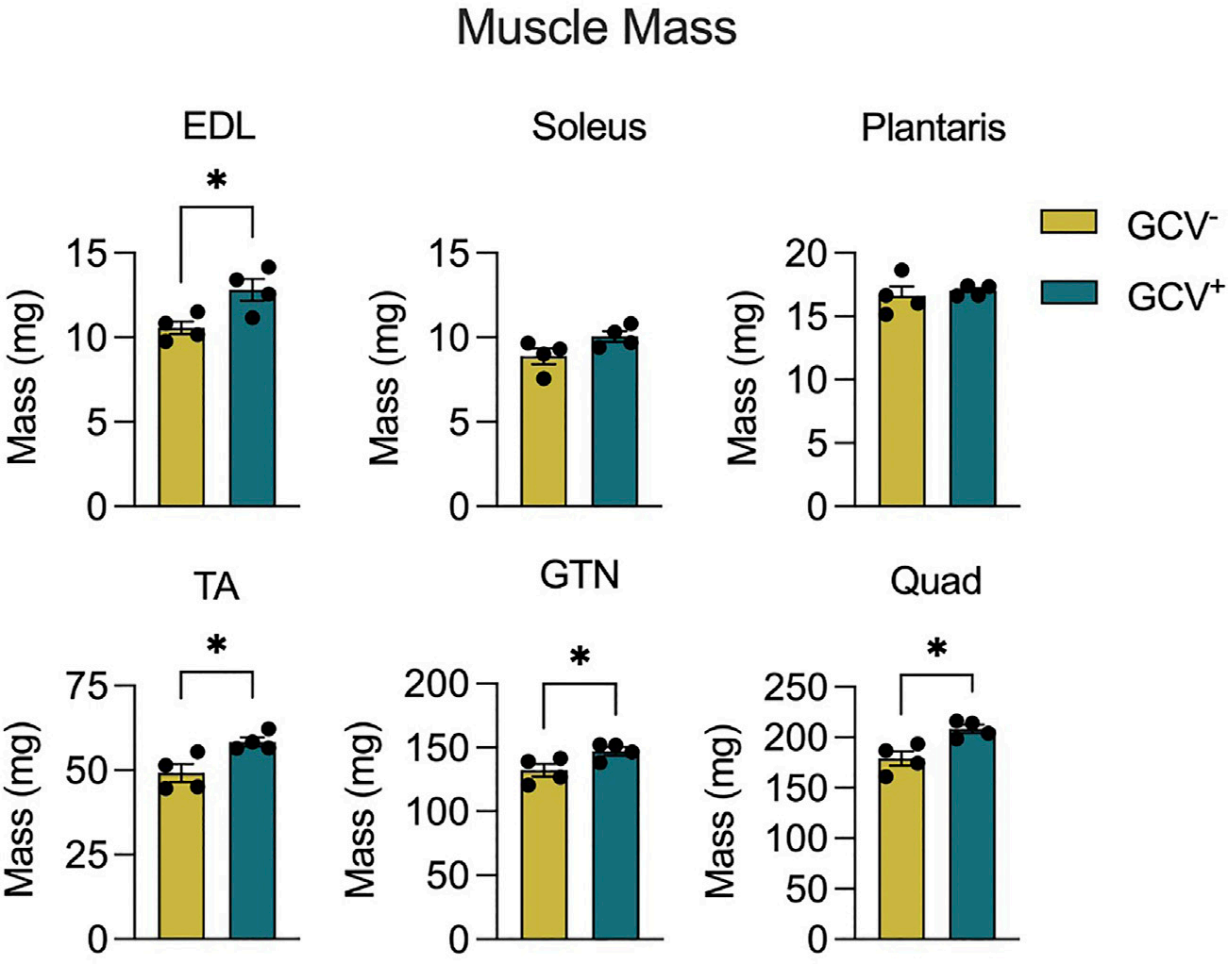
Hind limb muscle masses were preserved in male GCV^+^ mice. Masses are shown for extensor digitorum longus (EDL), tibialis anterior (TA), gastrocnemius (GTN) and quadriceps (Quad) muscles in milligrams for male ganciclovir treated (GCV^+^) and vehicle treated (GCV^−^) mice. Bars show the mean ± SEM of four mice per group with dots representing data from each individual male mouse. *Denotes *p* < .05 between groups by two-tailed unpaired t-test.

Body mass did not differ between GCV^+^ and GCV^−^ mice regardless of sex (Supplementary Figure 1), therefore, muscle masses are expressed in absolute terms, rather than normalized by body mass. Extensor digitorum longus (EDL), tibialis anterior (TA), gastrocnemius (GTN), and quadriceps (Quads) muscle masses were 12%, 19%, 11%, and 16% greater, respectively, in male GCV^+^ mice compared to vehicle controls (Figure 2), whereas plantaris and soleus muscle masses were not different between GCV^+^ and GCV^−^ mice. Despite the modestly higher muscle masses found in GCV^+^ compared with GCV^−^ mice, the effect on muscle mass was observed in the majority of the hind limb muscles examined, suggesting a broad effect to preserve muscle mass. In addition, the similarity in the degree of muscle mass preservation between muscles after late-life removal of p16 suggests that the effect is not specific to a particular muscle fiber composition. Despite broad effects in male mice, female GCV^+^ and GCV^−^ mice displayed no differences in mass for any of the muscles studied (Supplementary Figure 2).

**FIGURE 3:**
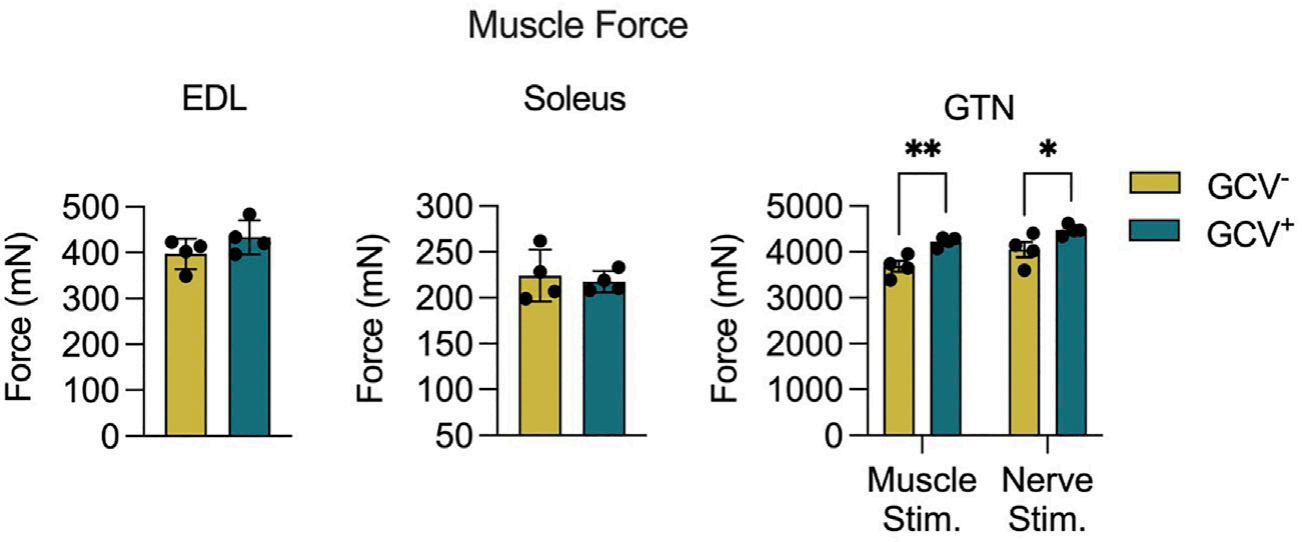
Gastrocnemius (GTN) muscles from male ganciclovir treated (GCV^−^) mice generated higher forces than muscles from vehicle treated (GCV^−^) mice. Data are shown for maximum isometric tetanic force (P_o_) generated by extensor digitorum longus (EDL), soleus and GTN muscles expressed in millinewtons (mN). EDL and soleus muscles were evaluated *in vitro*, while GTN muscles were evaluated *in situ* using both direct muscle stimulation and nerve stimulation. Bars show the mean ± SEM of four mice per group with dots representing data from each individual male mouse. *Denotes *p* < .05 and ** denotes *p* < .01 between groups by two-tailed unpaired t-test.

To determine the effects of p16^+^ cell depletion on muscle function we performed both *in vitro* and *in situ* muscle force testing. GCV treatment resulted in 13% and 10% greater maximum isometric tetanic force (P_o_) for GTN muscles with direct muscle stimulation and nerve simulation, respectively (Figure 3). These data are consistent with the higher GTN muscle mass for GCV^+^ mice and indicate that the increase in force generating capacity was attributable to an increase in functional muscle mass. Furthermore, specific forces (specific P_o_, sP_o_) did not differ between GCV^+^ and GCV^−^ mice (Supplementary Figure 3) indicating no effect of the removal of p16 expressing cells on the intrinsic force generating capacity of the muscle. We also found no alterations in the expression of acetylcholine receptor subunit alpha (AChRα) or muscle-specific kinase (MuSK), canonical neuromuscular junction related genes, between GTN muscles in GCV^+^ and GCV^−^ mice (Supplementary Figure 4). P_o_ was not different between GCV^+^ and GCV^−^ groups for EDL or soleus muscles from male mice (Figure 3) or for any muscles of female mice.

### CD68^+^ Positive Cell Numbers Are Reduced in EDL Muscles of GCV^+^ Mice

**FIGURE 4:**
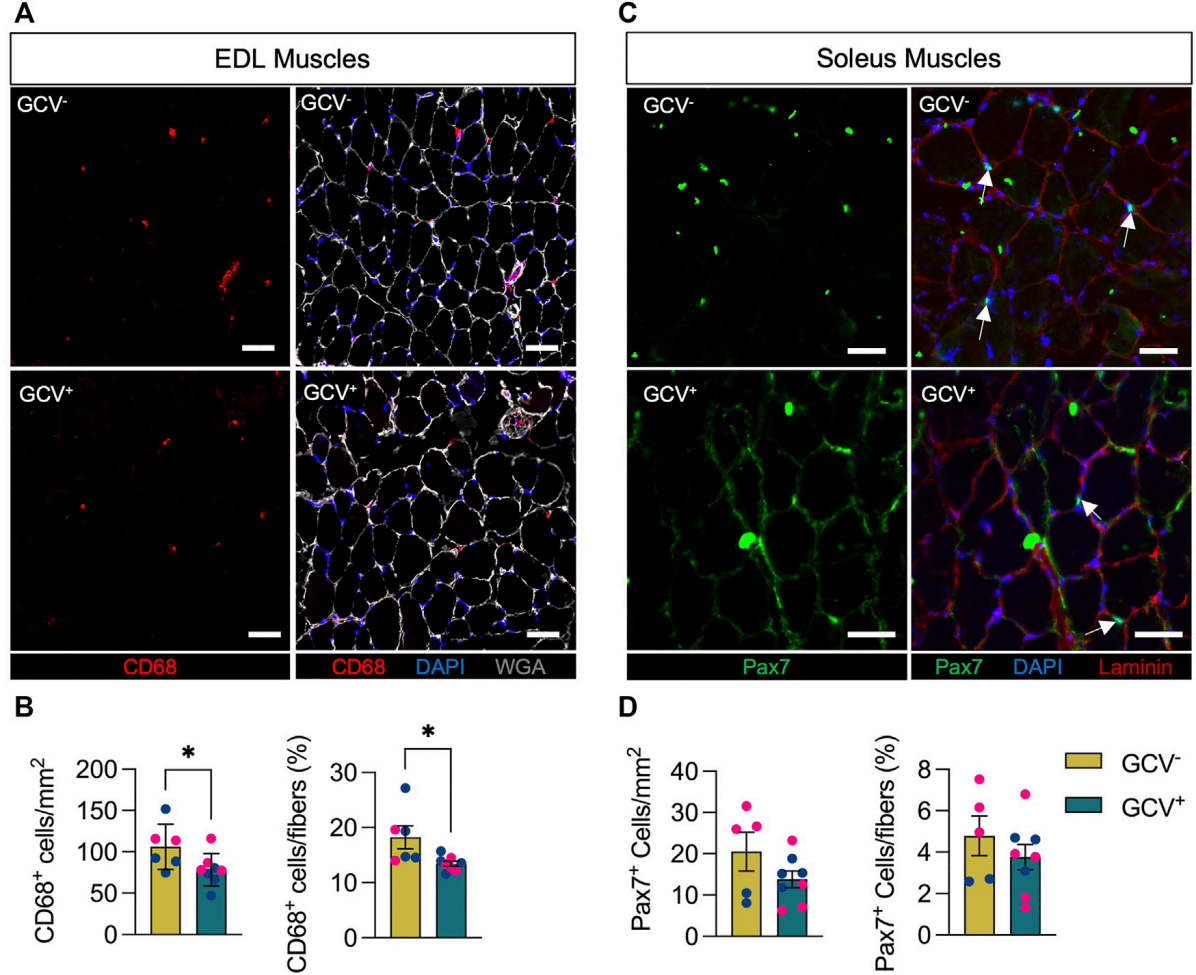
CD68-positive but not Pax7-positive cells were reduced in GCV^+^ mice. **(A)** Representative images are shown for cross sections of extensor digitorum longus (EDL) muscles stained with an antibody to the macrophage marker CD68 (red) as well as wheat germ agglutinin (WGA, white) and DAPI (blue). **(B)** Data showing counts of intramuscular CD68-positive cells are shown for cross sections from ganciclovir (GCV^+^) and vehicle treated (GCV^−^) mice. **(C)** Representative images are shown for cross sections of soleus muscles stained with an antibody to the satellite cell marker Pax7 (green) as well as laminin (red) and DAPI (blue). White arrows indicate Pax7-positive cells that overlap with DAPI and are between sarcolemma and basal lamina of muscle fibers. **(D)** Data showing counts of intramuscular Pax7-positive cells are shown for cross sections from ganciclovir (GCV^+^) and vehicle treated (GCV^−^) mice. For both CD68 and Pax7, cell counts are expressed either relative the area analyzed (mm^2^) or by the number of muscle fibers in the sections (%). Bars show the mean ± SEM of five to eight mice per group with dots representing data from each individual mouse. Blue and red data points represent male and female mice, respectively. *Denotes *p* < .05 between groups by two-tailed unpaired t-test. Scale bar = 50 μm.

Since the presence of senescent cells is thought to contribute to a pro-inflammatory environment, we assessed the total number of macrophages (CD68^+^ cells) in muscles of GCV^+^ and GCV^−^ mice. Compared with control GCV^−^ mice, EDL muscles of GCV^+^ mice showed a reduced number of intramuscular CD68^+^ cells. The magnitude of the difference was ∼25%, whether the number of cells were expressed relative to the cross-sectional area of the section or relative to the number of muscle fibers in the section (Figures 4A,B). We cannot establish from these experiments if the CD68^+^ cell depletion was due to genetic ablation of CD68^+^/p16^+^ cells following GCV treatment or due to a reduction in the inflammatory state of the tissue secondary to the loss of other senescent cell populations. Nevertheless, these data suggests that depletion of p16^+^ reduces intramuscular macrophage number in these aged mice and as such likely also impacted upon immune responses and function.

### Pax7^+^ Cell Content Is Not Impacted by Removal of p16-Expressing Cells

With growing evidence of increased cellular senescence markers in post-mitotic fibers (da Silva et al., 2019; Von Zglinicki et al., 2021) and increased expression of *p16* ^*INK4A*^ in resting geriatric and progeric muscle stem cells (Sousa-Victor et al., 2014), we chose to analyze the numbers of Pax7^+^ muscle stem cells (MuSC, satellite cells), which are critical regulators of the health and repair of skeletal muscle (Wang and Rudnicki, 2011; Abou-Khalil et al., 2015). We evaluated Pax7^+^ cells in cross sections of soleus muscles based on the higher prevalence of satellite cells associated with slow compared to fast muscle fibers with age (Verdijk et al., 2007; Verdijk et al., 2014). In contrast to the reduction in CD68^+^ macrophages following depletion of p16^+^ cells, the numbers of Pax7^+^ cells were not different between GCV^+^ and GCV^−^ mice whether expressed relative cross-sectional area or relative to the number of muscle fibers (Figures 4C,D).

### Plasma Inflammatory Factors and Muscle Cytokine Expression Are Minimally Impacted by p16-Positive Cell Depletion

**FIGURE 5:**
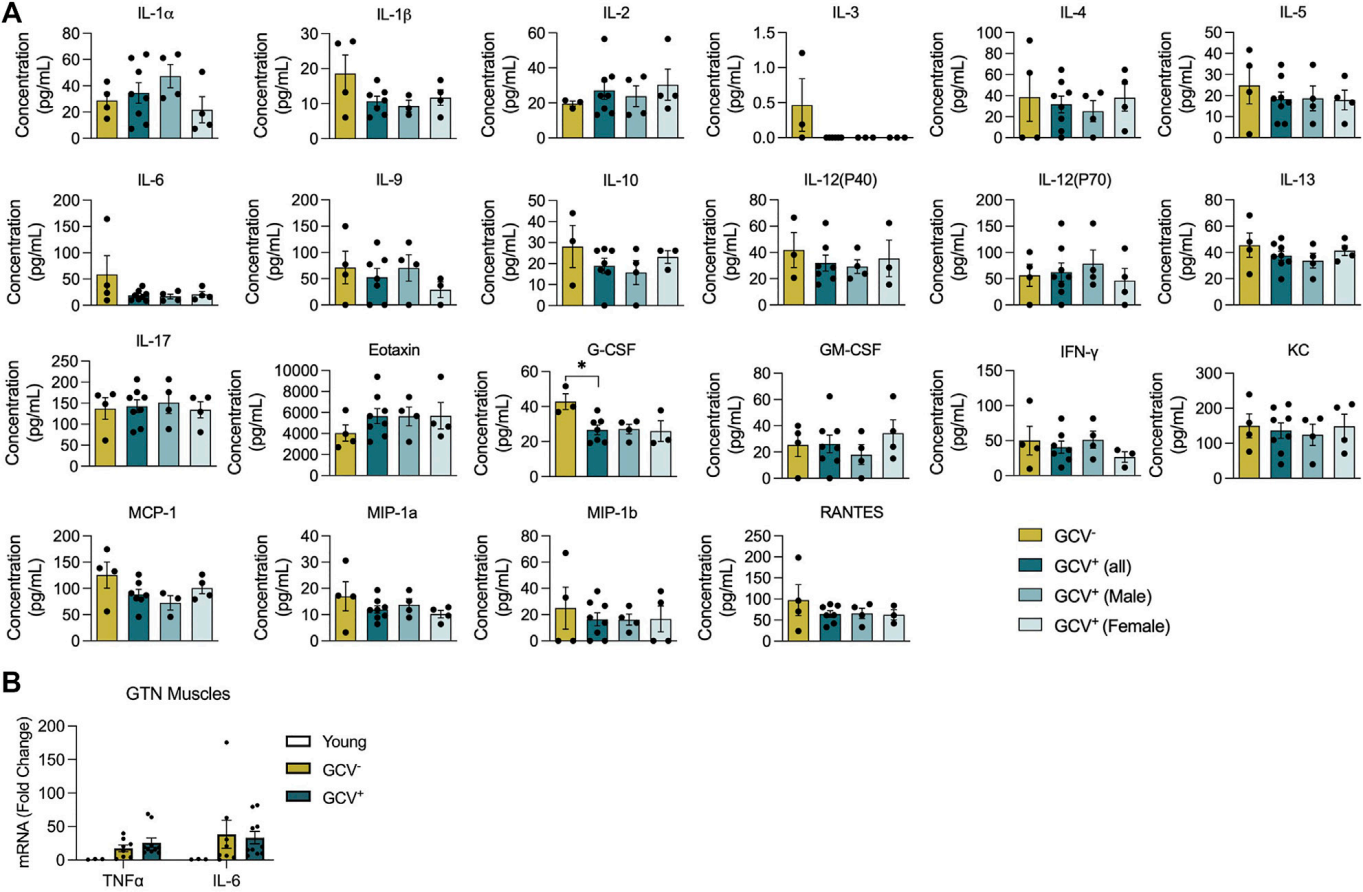
Plasma inflammatory factors and skeletal muscle cytokine mRNA expression were largely unaffected with p16*-*positive cell deletion. **(A)** Plasma protein levels (pg/ml) for 22 inflammatory mediators are shown for saline treated controls (GCV^−^), combined male and female GCV treated mice, GCV^+^ (all), male GCV treated mice, GCV^+^ (Male), and female GCV treated mice, GCV^+^(Female). **(B)** TNFα and IL-6 mRNA expression relative to 18s mRNA in gastrocnemius (GTN) muscles were not significantly different in young, GCV^−^, and GCV^+^ mice. Bars show the mean ± SEM of three to eight mice per group with dots representing data from each individual mouse. *Denotes *p* < .05 between groups by one-way ANOVA.

Since GCV treatment in p16-3MR mice results in whole-body depletion of p16^+^ cells in tissues that are accessible to GCV treatment, we assessed plasma profiles for a wide array of inflammatory mediators and mRNA levels for select cytokines from skeletal muscle isolates. Out of a panel of 22 inflammatory mediators assessed in plasma, only granulocyte colony-stimulating factor (G-CSF), to be lower in GCV^+^ mice compared to GCV^−^ controls (Figure 5A). Furthermore, mRNA expression of tumor-necrosis factor alpha (TNF-α) and interleukin-6 (IL-6) mRNA, were not modified in GTN muscles of GCV^+^ mice (Figure 5B). These data suggest that alterations in inflammatory gene and protein expression are not largely affected after a late-life 5-month long p16^+^ cell depletion regimen.

## Discussion

Herein we report the effects of late-life removal of p16^+^ cells on skeletal muscle function. Our study shows that modest muscle mass and muscle force preservation can be achieved with genetic ablation of p16^+^ cells in male mice. The degree of muscle mass (+11%) that was preserved in the GTN muscles with GCV treatment closely reflected the degree of increased force generation (+11.5%). These findings suggest that the mass preservation achieved through elimination of p16^+^ expressing cells was in fact preservation of contractile tissue with no change in inherent contractility, a conclusion supported by no effect of GCV treatment on specific force (force/CSA). Since GCV treatment results in whole-body removal of p16^+^ cells, the preservation in mass is likely a result of systemic influences on the skeletal muscle tissue rather than caused by direct changes to skeletal muscle fibers. TA, EDL, and quadriceps muscles also showed preservation of mass in GCV^+^ mice. We did not assess force generating capacity in TA or quadriceps muscles, and although differences in EDL muscles did not reach statistical significance, the trend was for an increase in treated mice when compared with saline treated controls did not reach statistical significance. Despite the similarity in force generation, increased muscle mass provides additional benefits as skeletal muscle serves other functional roles such as acting as a protein repository and as a site for glycolipid metabolism. Therefore, the preservation of muscle mass could provide other metabolic health benefits.

The current study utilized a treatment regimen that spanned many months, and muscles were evaluated at the most advance stages of life. It is possible that benefits in muscle function through deletion of p16^+^ cells were achieved earlier during the treatment protocol. However, direct rigorous assessments of isolated muscle function are terminal procedures and as such we were unable to longitudinally assess the effects of GCV treatment on muscle function. Our findings of some evidence of preservation of muscle masses and strength were consistent with the report by Xu et al. (2018) of greater maximal walking speed, hanging endurance, grip strength, treadmill endurance and daily activity in mice as a result of late-life treatment with a senolytic cocktail of dasatinib plus quercetin. Since senolytics work through disabling senescent anti-apoptotic pathways (SCAP), a distinct mechanism of action compared to genetic ablation, our method only specifically removes p16^INK4A^ expressing cells and does not directly affect other senescent promoting pathways such as p53/p21^WAF1/CIP1^ (Kumari and Jat, 2021). Nevertheless, our data show that directly attenuating the *p16* ^*INK4A*^ pathway also results in modest skeletal muscle benefits in male mice.

An interesting finding in our study was the sexual dimorphism observed for our muscle assessments. The significant protective effect observed for muscle mass in male mice by the elimination of p16^+^ cells was not seen for GCV^+^ female mice. A recent study in mice showed that male mice, at 20-month of age, showed higher expression of both p16 and p21 mRNA in various tissues compared to female mice (Yousefzadeh et al., 2020). However, at 30-month of age the numbers of senescent cells were equivalent between both sexes. Considering our treatment regimen began at 20-month of age, differences in the level of p16 expressing cells in male compared with female p16-3MR mice at this time-point may potentially explain the greater benefit of the deletion of p16^+^ cells observed for male mice. Although sarcopenia is equally prevalent in both men and women, the rate of muscle mass and strength decline has also been reported to be faster in men (Mitchell et al., 2012). Therefore, understanding the sex specific mechanisms that drive sarcopenia are important for advancing therapeutics that promote muscle health benefits.

Tissue resident macrophages are critical for skeletal muscle regeneration after injury and muscle regeneration after injury requires inflammatory signals for proper repair (Tidball and Villalta, 2010; Chazaud, 2020). Furthermore, other studies have found that removal of senescent cells at injury sites has a negative impact on tissue regeneration (Yun, 2018; Da Silva-Álvarez et al., 2020). Therefore, whether the observation in the present study of a reduction in intramuscular CD68^+^ macrophages with p16^+^ cell ablation provides a functional benefit to muscle tissue remains unclear. Specifically, the effect of the reduction in CD68^+^ cells and perhaps other p16-expressing cells on the ability to respond to insults such as nerve or muscle injuries remains to be established. However, macrophage number has been shown to increase with age and produce low grade inflammation that contributes to muscle mass and functional decline (Wang et al., 2015). Therefore, our finding of reduced macrophage number with GCV^+^ may provide a beneficial response in basal conditions. Moreover, another recent study showed that absence of fibro-adipogenic progenitor (FAP) senescence after exercise leads to muscle degeneration with FAP accumulation (Saito et al., 2020). One benefit to removing cellular senescence is improvement of muscle stem cell function. At baseline, muscle stem cells from geriatric and progeric models have been shown to display an upregulation of senescence-associated genes including *p16* ^*INK4A*^ and a reduced regenerative capacity upon muscle injury (Sousa-Victor et al., 2014). Repression of *p16* ^*INK4A*^ through shRNA restores quiescence and regeneration in aged mice. Our study showed no overall change in the number of Pax7^+^ muscle stems cells in GCV^+^ mice; however, we did not confirm whether the muscle stem cells also co-expressed *p16* ^*INK4A*^ or if there was an effect of clearing p16^+^ cells on regenerative capacity. Future studies should investigate the critical senescent cell types that provide the greatest therapeutic efficacy when removed or modified.

The SASP phenotype is one of the most extensively studied features of cellular senescence (Di Micco et al., 2021). Our group sought out to understand whether removal of p16^+^ cells through a 5-month long treatment program would reduce systemic cytokine expression. We therefore assessed plasma chemokine inflammatory profiles using a 22-factor array. Our experiments showed no difference in overall inflammatory profiles between saline treated controls (GCV^−^) and GCV^+^ mice (both male and female). Although somewhat surprising, these data are consistent with findings from two independent studies that found no change in serum/plasma cytokine levels with p16^+^ cell ablation and treatment with senolytics (Farr et al., 2017; Dungan et al., 2021). Similarly, we found no differences in expression of TNFα or IL-6 mRNA between GCV^+^ and GCV^−^ GTN muscles. These data conflict with Baker et al. (2016), who found significantly reduced levels in both TNFα and IL-6 mRNA in GTN muscles of mice with removal of p16^+^ cells. However, their study used a different model of p16^+^ cell ablation and evaluated mice at a much younger age, with treatments to remove p16^+^ cells beginning at 12 months of age and evaluations performed at 18 months. Our approach in the current study of deleting p16^+^ cells starting at 20-month of age and evaluating mice at the significantly more advanced ages of greater than 26 months may explain the difference between the present findings and those of Baker et al. Further investigation is required to understand the efficacy window for clearing p16^+^ cells to promote muscular function.

Overall, our data show that elimination of *p16* ^*INK4A*^ expressing cells can provide modest late-life functional improvements and muscle mass preservation. These changes, however, are sex-specific and therefore warrant further examination into the molecular processes that induce muscle health benefits in both male and female. Future studies are needed to determine molecular targets and treatment windows to combat overall skeletal muscle aging.

### Study Approval

All animal experiments were approved by the University of Michigan Institutional Animal Care and Use committee (IACUC) (PRO00008744).

## Data Availability

The raw data supporting the conclusions of this article will be made available by the authors, without undue reservation.
