# Supplementary material for: Removal of p16 INK4 Expressing Cells in Late Life has Moderate Beneficial Effects on Skeletal Muscle Function in Male Mice
Source: Front Aging. 2022 Jan 26;2:821904. doi: 10.3389/fragi.2021.821904 (PMC9261355; doi:10.3389/fragi.2021.821904)
Supplement: Supplementary file 1 [file DataSheet1.docx]

**Supplementary Materials**


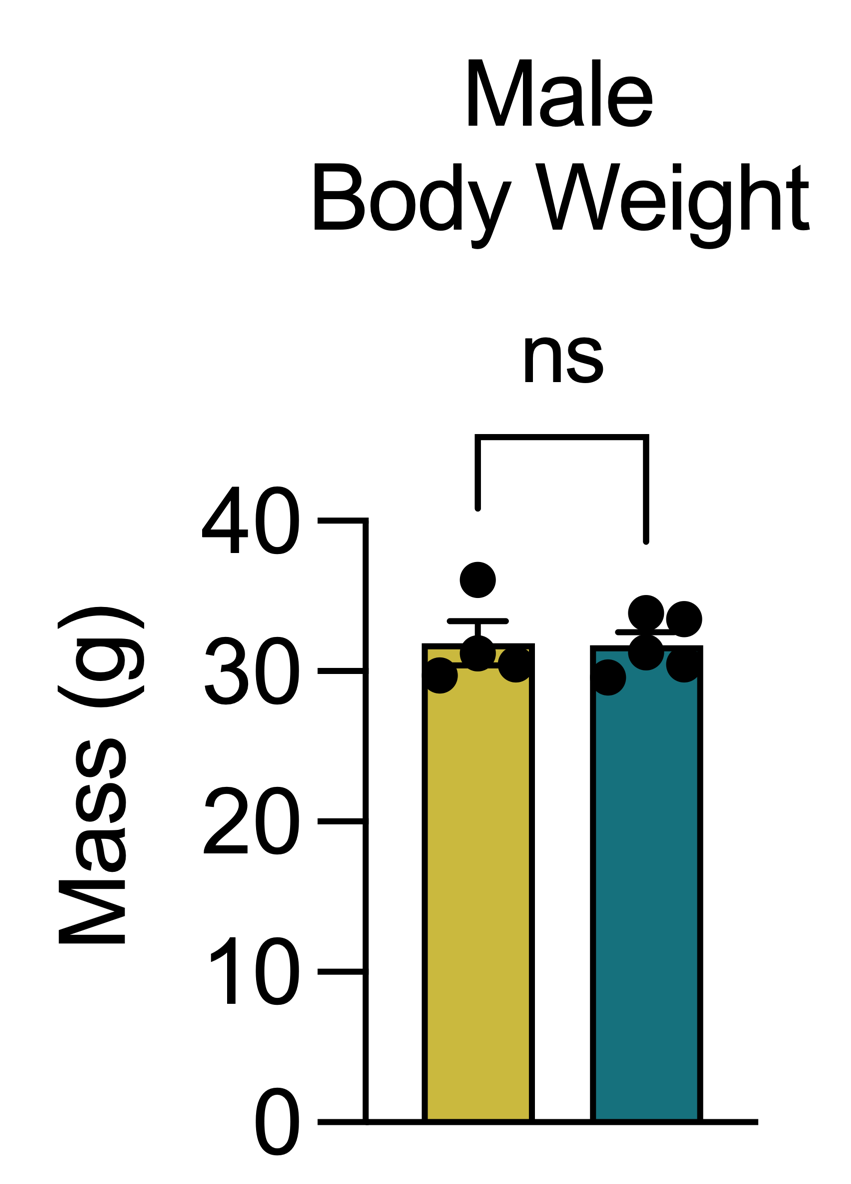

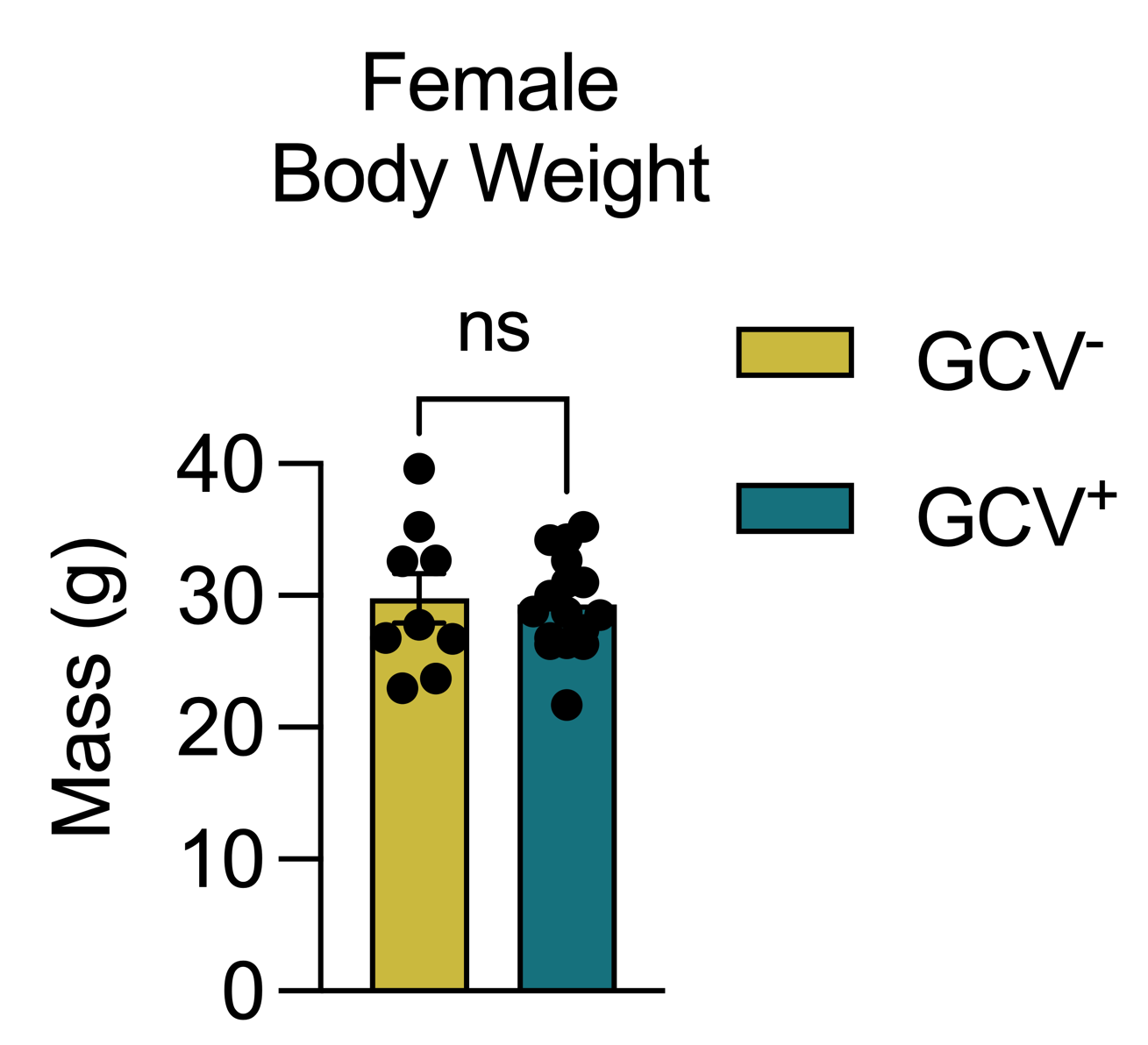


**Supplementary Material – Figure 1.** Body mass did not differ between GCV^+^ and GCV^-^ mice. Bars show the mean ± SEM of 4-16 mice per group with dots representing data from each individual mouse. NS denotes *p-*value > 0.05 between groups by two-tailed unpaired *t*-test.

**Supplementary Material – Figure 2.** Absolute muscle mass and force did not differ between female GCV^+^ and GCV^-^ mice. (A) Extensor digitorum longus (EDL), soleus, plantaris, tibialis anterior (TA), gastrocnemius (GTN) and quadriceps (Quad) muscle masses were not significantly different between GCV^+^ and GCV^-^ mice. (B) Similarly, EDL, soleus, and GTN absolute muscle forces were not significantly different. Bars show the mean ± SEM of 5-16 mice per group with dots representing data from each individual mouse.


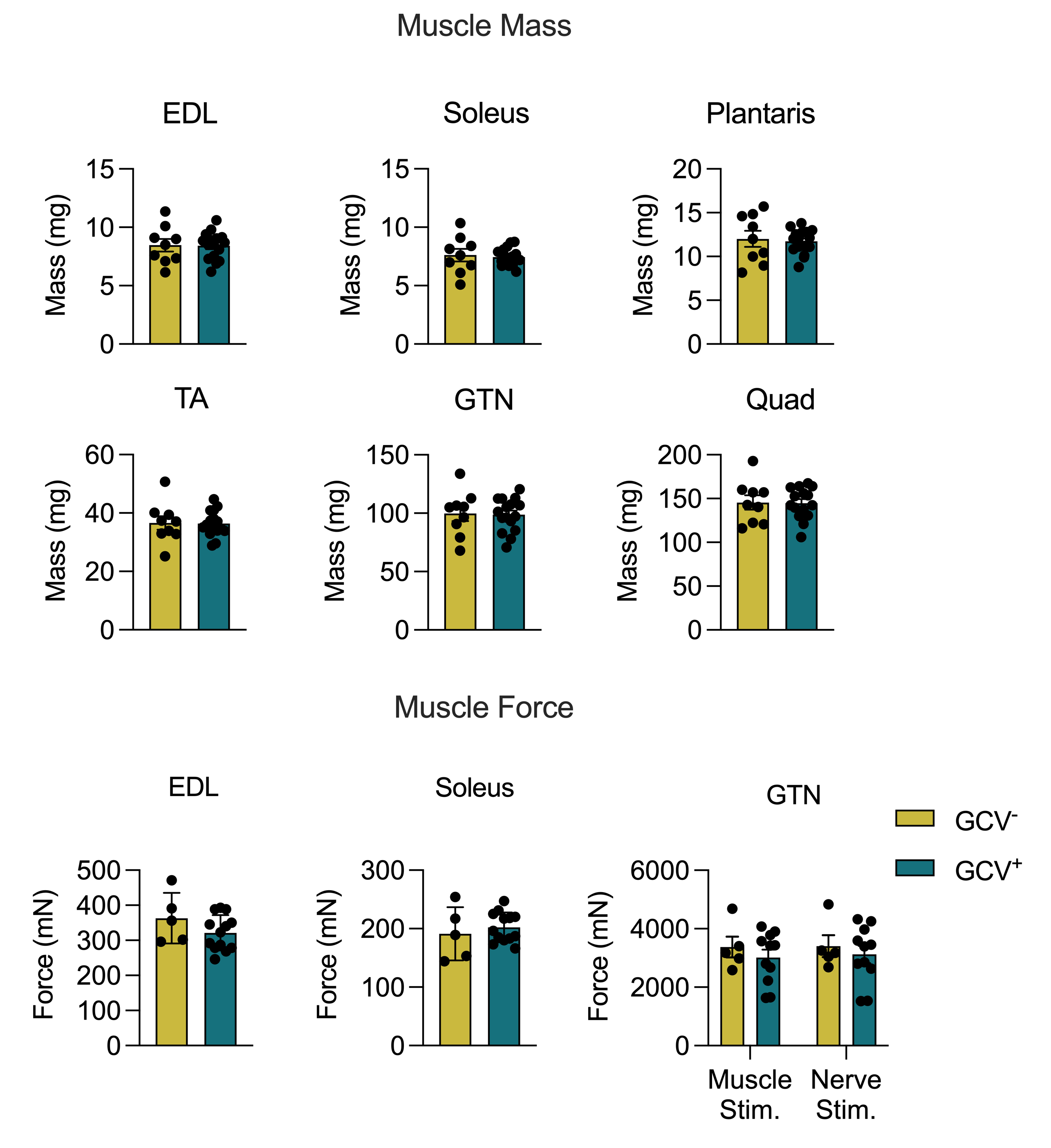


A

B


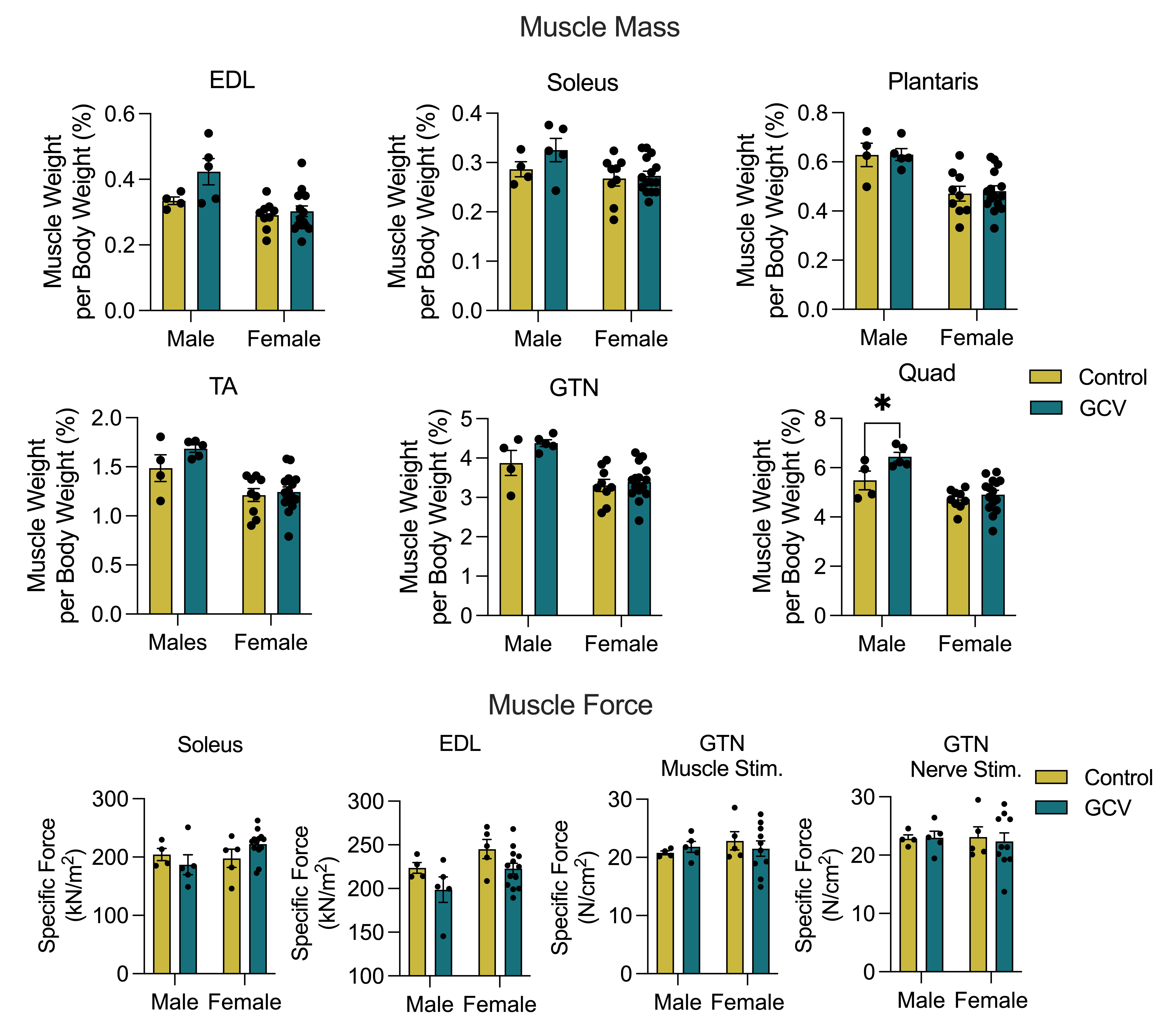


**Supplementary Material – Figure 3.** Specific forces did not differ between GCV^+^ and GCV^-^ mice, regardless of sex. Soleus, EDL, and GTN specific muscle forces were not significantly different. Bars show the mean ± SEM of 5-16 mice per group with dots representing data from each individual mouse.

**Supplementary Material – Figure 4.** AChRα and MuSK mRNA expression in gastrocnemius muscles were not significantly different in young, GCV^-^, and GCV^+^ mice. Bars show the mean ± SEM of 3-8 mice per group with dots representing data from each individual mouse.
